# Editorial: Public health policies for improved oral health outcomes

**DOI:** 10.3389/froh.2026.1828750

**Published:** 2026-04-28

**Authors:** Praveen Jodalli, Mamata Hebbal, Roberto Ariel Abeldaño Zuñiga, M Shodan

**Affiliations:** 1 Department of Public Health Dentistry, Manipal College of Dental Sciences Mangalore, Manipal Academy of Higher Education, Manipal, India; 2 Community Dentistry Division, Preventive Dental Sciences Department, College of Dentistry, Princess Nourah Bint Abdulrahman University, Riyadh, Saudi Arabia; 3 Faculty of Health Sciences University of Eastern Finland, Helsingin Yliopisto, Kuopio, Finland; 4 Department of Public Health Dentistry, SDM College of Dental Sciences & Hospital, Sattur, Dharwad, India

**Keywords:** equity, oral health, oral health policies, public health policies, sustainability

## Introduction

Oral health is a significant component of overall health, quality of life, and well-being; however, it is frequently overlooked in global public health policies. The existence of gaps in oral health across socioeconomic categories and geographic regions emphasises the critical need for comprehensive public health policies that address prevention, early detection, enhanced access to care, equity, and, most significantly, sustainability. This editorial provides a comprehensive narrative on the significance of strengthening public health policies for improved oral health outcomes at a population level and also discusses the multifactorial nature of oral health by outlining its social determinants, behavioural risk factors, impact on vulnerable populations, innovations, and sustainability for healthcare delivery. Furthermore, this editorial discusses the importance of integrating oral health into broader public health practices and repositioning oral health as a critical component of universal health coverage.

### Determinants of oral health

Global epidemiological studies have shown that oral diseases exert a significant burden on healthcare systems and communities (Ma et al.; Kateeb). The burden of orofacial clefts from 1990 to 2021 at global, regional, and national levels reported that the burden of orofacial clefts remains a major public health challenge globally. This study emphasises the need for stronger preventive health policies that address congenital oral abnormalities alongside other common oral health diseases (Ma et al.). Beyond disease burden, oral health is influenced by socioeconomic background, culture, geography, and historical context. A case study of an oral health promotion programme in New Caledonia demonstrated how oral health promotion can be utilised not just to overcome barriers, but also to address health disparities (Skandrani et al.). The findings highlight the need for oral health strategies that incorporate education, community participation, and broader health determinants (Skandrani et al.). Another study, on the associations between sexual identity and caries risk indicators among adolescents and adults in Nigeria*,* suggested that oral health strategies must address stigma, discrimination and disadvantaged and marginalised populations (Folayan et al.). Ensuring equity must therefore remain a central pillar of oral health policies.

### Behavioural risk factors of oral health

Public health policies must also address behavioural risk factors associated with oral disorders. Excessive sugar consumption, a substantial modifiable risk factor for oral disorders, is frequently ignored. Fiscal policies, such as food taxes on sugar-sweetened beverages, have been implemented as part of national public health policies to regulate consumption. Evidence from a systematic review on the effectiveness of sugar taxation policies in Asia and Africa demonstrated changes in the volume of purchase, consumption, and sugar content of taxed items, in addition to a favourable impact of sugar-sweetened beverage taxes on reducing taxed item consumption (Fernandes et al.). These findings show the benefit of regulatory measures in promoting healthy behaviours.

### Systemic and biological dimensions of oral health

In addition to behavioural risk factors, emerging biological investigations have shown the significance of systemic factors in oral health. A study exploring the interplay of vitamin D, salivary antimicrobial peptides, and cytokines in oral immunity and disease prevention reported a possible relationship between metabolic syndrome and poor oral health, as demonstrated by higher caries incidence and periodontal involvement (Tumkur Shivakumar et al.). Understanding these biological linkages is essential for improving future preventive approaches and integrating healthcare interventions that address both systemic and oral health concerns.

### Oral health in vulnerable and underserved populations

Certain populations require particular attention within oral health policy frameworks. Paediatric oral health is frequently isolated from general child health services. Paediatricians, who are typically the initial point of contact for families, receive minimal oral health training, whereas paediatric dentists mainly encounter children when diseases have progressed, limiting opportunities for preventive and early care. A study on policy integration of paediatric oral health reported structural challenges such as fragmented professional education, ineffective referral networks, insufficient insurance, and inconsistent access to preventive services, which hinder progress (Mistry et al.). Families face significant out-of-pocket expenses, especially in low- and middle-income countries, where preventive oral care is excluded from many paediatric health plans. A scoping review of global policy approaches to combatting early childhood caries highlighted the urgent need to include paediatric oral health in universal health coverage and child health strategies, which will increase equity and sustainability (Lydia et al.). Urgent, comprehensive, equity-orientated, and system-integrated policy interventions are needed to effectively prevent paediatric oral diseases. Similarly, displaced populations, especially refugees and internally displaced persons, bear a disproportionate burden of untreated oral diseases. A study on the social determinants of oral health in situations of conflict observed that conflict and forced displacement exacerbate social determinants of health, transforming food insecurity, limited access to safe water, and the collapse of healthcare facilities into acute risk factors for oral disease (Kateeb). Even in fragile circumstances, low-cost, minimally invasive procedures such as daily oral hygiene support, fluoride use, and basic emergency dental interventions can be implemented. This Perspective demands the urgent recognition of oral health as a critical aspect of humanitarian health policy and practice. Positioning oral health within humanitarian frameworks is both possible and ethically imperative.

### Innovations in oral health policy and practice

Innovation plays a vital role in policymaking, but it needs to be handled carefully. For example, the DN (dynamic navigation) PUBLIC framework for enhanced oral healthcare precision presented dynamic navigation as a technology capable of improving precision, reducing problems, and increasing access to advanced oral care when combined with appropriate regulation, training, and equitable implementation. Public health policies could impact whether innovation increases or reduces inequalities (Bhalerao and Kumar).

### Integration of oral health into public health initiatives

Integrating oral health into broader public health initiatives is another key policy priority. A study of the National Oral Health Programme and National Tobacco Control Programme in India suggested that integrating tobacco control programmes into oral health policies is a successful public health strategy. Policy coherence can help to support integrated awareness campaigns, screening programmes, and coordinated surveillance systems (Pai et al.). Another study on adolescent- responsive oral, mental, sexual, and reproductive healthcare services in Africa through dental clinics suggested integrating oral health with mental, sexual, and reproductive healthcare services via dental clinics (Sam-Agudu et al.). These are not isolated innovations; rather, they reflect a broader policy approach in which oral health is linked to other goals rather than being administratively segregated. Hungary's primary care dental clusters serve as an example of how proactive professional collaboration within dental clusters can enhance oral health infrastructure, increase population access to preventive dental care and improve service quality (Sztrilich et al.). These types of approaches demonstrate how a supportive policy framework can enhance collaboration between healthcare providers, improve service delivery, and align with the common risk factor approach in public health.

### Sustainability in oral health policies

Sustainability is an important part of oral health policies. A review of sustainable practices reported that they ensure preventive and therapeutic services remain accessible and affordable, aligning with broader global health goals, such as the United Nations Sustainable Development Goals (Guerra et al.). In addition, an article on oral health outcomes through public health policy reform suggested that integrating the pillars of sustainability, equality, and targeted interventions to combat the burden of oral health diseases provides a solid foundation for developing public health policies and programmes (Anyikwa and Ogwo).

In conclusion, improving population-level oral health outcomes necessitates structured, comprehensive public health policies that incorporate preventive measures, early identification, increased access to healthcare, inclusivity, equity, innovation, collaboration, multisectoral strategies, and sustainability. To put this into practice, separate policy initiatives are necessary. Oral healthcare should be included as a vital component of Universal Health Coverage (UHC) packages to ensure equitable access to oral health care. Fiscal policies, such as taxes on sugar-sweetened beverages, tobacco, and alcohol, should be implemented to address modifiable risk factors. Strengthening primary healthcare facilities by incorporating oral health screening, a structured referral pathway, and training and capacity building for non-dental healthcare providers can help with early detection and prompt management. Additionally, community and school-based activities should be encouraged to promote preventive care. Finally, multisectoral collaboration is necessary to position oral health policies in a broader interface with other non-communicable disease frameworks, ensuring a more integrated and sustainable approach to improving oral health outcomes.

